# Difficult to Treat Psoriatic Arthritis: The Road So Far

**DOI:** 10.31138/mjr.2506241.dtt

**Published:** 2024-12-31

**Authors:** Angeliki E. Dimopoulou, Konstantinos D. Vassilakis, George E. Fragoulis

**Affiliations:** Joint Academic Rheumatology Programme, First Department of Propaedeutic and Internal Medicine, National and Kapodistrian University of Athens, Athens, Greece

**Keywords:** psoriatic arthritis, difficult to treat, comorbidities, bDMARDS

## Abstract

Psoriatic Arthritis (PsA) is a multifaceted, immune-mediated disease marked by chronic musculoskeletal inflammation (peripheral arthritis and axial disease, dactylitis, and enthesitis), extra-musculoskeletal manifestations (psoriasis, nail involvement, Inflammatory Bowel Disease [IBD], and uveitis) and multi-comorbidity (cardiovascular disease, metabolic syndrome, mental health disorders, and fibromyalgia). Immunological and non-immunological factors have led, despite the progress made in the understanding, treatment and management of PsA, to a minority of patients being able to achieve satisfactory outcomes. Following the establishment of the definition for difficult to treat rheumatoid arthritis, efforts are underway for difficult to treat PsA (D2T PsA). Defining D2T PsA and its predictors is crucial for advancing clinical trials, treatment strategies, and patient care. Proposed definitions and criteria for D2T PsA vary, but the few available data indicate that extensive psoriasis, axial involvement, obesity, female gender, and comorbidities like IBD, depression, and fibromyalgia are involved. Concerns are also raised for the lack of a universally accepted index for disease activity measurement and for the inclusion of a time-related criterion in the definition of D2T. Moreover, the potential need for distinction between D2T and refractory-to-treatment PsA has also been suggested. In this narrative review, we summarise the current knowledge on the D2T PsA field, highlighting the gaps and the necessity of the “D2T” concept, providing further considerations on the matter.

## INTRODUCTION

Psoriatic Arthritis (PsA) is a complex, immune-mediated disease, characterised by chronic musculoskeletal inflammation. The clinical picture of this disease is widely heterogeneous, with multiple tissues being involved. Peripheral arthritis, axial disease, dactylitis and enthesitis are classic musculoskeletal manifestations.^1^ However, the complexity of PsA’s disease profile lies in the additional extra-musculoskeletal associations and its multiple recorded comorbidities. Extra-musculoskeletal manifestations include psoriasis, nail involvement, Inflammatory Bowel Disease (IBD) and eye involvement, most commonly uveitis.^2^ Simultaneously, amongst the individuals with PsA there is a high prevalence of cardiovascular disease, metabolic syndrome, mental health disorders (depression, anxiety, sleep disorders, mood disorders) and fibromyalgia.^3,4^ In an effort to encompass this multi-faceted clinical picture the terms “psoriatic disease”, or “psoriatic syndrome” have been proposed.^5,6^

Over the last years, we have gained a deeper understanding of the pathogenetic mechanism of this disease. Genetic and environmental factors (especially the microbiome), along with mechanical stress, have been incriminated as causative agents. Key role has been attributed to the interleukin (IL)-23/IL-17 axis, a pathway that also involves other cytokines, like Tumour Necrosis Factor (TNF) and IL-22.^5,7^

The aforementioned progress noted in the comprehension of PsA has led to the enrichment of its treatment armoury with multiple new treatment agents. Apart from conventional synthetic (cs) disease modifying anti-rheumatic drugs (DMARDs), novel drugs, tailored to target the immunological pathogenetic mechanism that has been implicated, have been approved and have become available. Namely: the biological (b) DMARDs, targeting the TNF and the IL-23/IL-17 axis and the targeted synthetic (ts) DMARDs that inhibit Janus kinases (JAKs) or phosphodiesterase 4 (PDE4).^7^ As a result, the choice of agent is better adjusted to each patients’ mosaic of disease manifestations, potential contraindications, and previous treatment strategies, often after multidisciplinary collaboration, with the main goal being to ameliorate health related Quality of Life (QoL) and to eliminate inflammation.^8^

Nevertheless, even with the plethora of available drugs and the greater personalisation of treatment regimen to the patients’ profile, desired treatment outcomes are not achieved in a satisfactory percentage of the patients. For example, in a recent real-life, contemporary, cross-sectional, multicentre, nationwide study that enrolled 923 patients, Minimal Disease Activity (MDA) was achieved in less than half of the enrolled patients (60% was treated with bDMARDs).^9^ This is in accordance with the results of a meta-analysis, in which an overall 37% (95% CI 34%–41%, I2 = 93%) of patients, in the selected real-life studies, achieved MDA.^10^

In 2021, the “difficult-to-treat” (D2T) terminology was first coined, describing difficult to treat rheumatoid arthritis (RA). The European Alliance of Associations for Rheumatology (EULAR)’s task force agreed upon the definition of D2T RA,^11^ it being: active disease after ≥2 b/tsDMARDs with different mechanisms of action (MOAs), with prior failure of csDMARD therapy (unless contra-indicated). At least moderate disease activity (MODA), signs and symptoms of RA, inability to taper glucocorticoid treatment, rapid radiographic progression and well-controlled disease but still low quality of life due to symptoms, were all considered signs of active disease. The rheumatologist’s and/or patient’s perception of the degree of management achieved was also suggested as a criterion. Motivated from that, the PsA community is now on the process of consolidating a definition of D2T PsA.

### Studies examining D2T PsA

For this narrative review, we searched PubMed to include studies examining the D2T PsA concept, using the following keywords: “Psoriatic arthritis” AND “difficult to treat”, OR “refractory”. Thus far, less than a handful of studies were found to have been published on this subject:

Firstly, in 2022, Perrotta et al.^12^ conducted a retrospective study in which 106 patients were enrolled and D2T PsA was defined as failure of at least 2 b/ts DMARDs, with different MOAs, after failing cs DMARDs (unless contraindicated), with demonstration of signs of active, or progressive disease and perception of management as problematic by the rheumatologist and/or the patient (Table 1). Patients were considered to have active or progressive disease having at least one of the following: at least moderate disease activity according to validated measures (e.g. joint count, Disease Activity in PsA>14, not reaching MDA), signs (e.g. acute phase reactants, imaging) and symptoms (e.g. joint-related) of active disease, rapid radiographic progression, or PsA symptoms that lead to reduced QoL. In a univariate analysis, they found that D2T patients had more frequent skin involvement, higher incidence of fibromyalgia, significantly higher BMI (Body Mass Index) and they scored a higher BSA (Body Surface Area), DAPSA (Disease Activity Score for Psoriatic Arthritis), HAQ-DI (Health Assessment Questionnaire – Disability Index), PsAID (Psoriatic Arthritis Impact of Disease), PtGA (Patient’s Global Assessment) and VAS (Visual Analogue Scale) pain assessment scores and lower rate of PASS (Patient Acceptable Symptom Scale). They also documented higher median FCI (Functional Comorbidity Index), with hypertension, dyslipidaemia, metabolic syndrome, Diabetes Mellitus, cardiovascular disease, osteoarthritis and anxiety/depression being the most common comorbidities and a significant delay between diagnosis and initiation of first ts/b DMARD.

**Table 1.** Demographic and clinical characteristics of the included studies.

**First author – Year**	**Number of patients (Classified as D2T)**	**D2T PsA Definition**	**Main findings**				**Limitations**
Perrotta et al. 2021^12^	106 (36)	All 3 criteria must be present: - failure of ≥2 b/ts DMARDs with different MOAs after failing csDMARDs (unless contraindicated) - signs of active disease/progressive disease* - management deemed problematic by rheumatologist and/or patient	Features	nD2T	D2T	p	- Not adjusted for potential confounders - Small sample size - No data for b/tsDMARDs andcsDMARD co-administration
			BMI, median	25.7	27.7	0.032	
			Psoriasis^1^ , n	35/65	27/32	<0.001	
			BSA, median	0.25	1	<0.01	
			PASS yes, n	66/70	8/36	<0.01	
			PtGA, median	2	6	<0.01	
			Pain on VAS, median	2.5	7	<0.01	
			DAPSA, median	7.1	17.2	<0.01	
			PsAID, median	1.91	5.30	<0.001	
			HAQ-DI, median	0.25	1	<0.001	
			FCI, median	0	1	0.021	
			FCI≥1^˚^, n	25/69	21/36	0.030	
			Fibromyalgia, n	5/69	8/36	0.022	
			Time from diagnosis to first b/tsDMARDs, median months	24	54	<0.01	
			≥1~	30/70	36/36	<0.01	
			≥2~	6/70	36/36	<0.01	
			≥3~	3/70	24/36	<0.01	
Philippoteaux et al. 2023^13^	150 (49)	Patients who failed ≥2 b/ts DMARDs with different MOAs	Feature of D2T patients	OR (95% CI)		p	- Small sample size - Socioeconomic characteristics were not captured - No data for b/tsDMARDs and csDMARD co-administration
			Axial involvement^3^	2.37 (1.06 - 5.69)		0.035	
			Peripheral structural damage^3^	2.57 (1.16 – 5.69 )		0.020	
			bDMARD discontinuation due to poor dermatological control	2.99 (1.32 – 6.74 )		0.008	
Vassilakis et al. 2024^14^	467 (77)	Main Definition: ≥ 6 months of disease duration + failed to cs ≥1 DMARD (unless contraindicated) and ≥2 ts/b DMARDs (except for apremilast) with different MOAs + had either at least MODA (DAPSA >14 ) and/or were not in MDA at the time of their assessment	Feature of D2T patient	OR (95% CI)		p	
			BSA>3%^×^	5.05 (2.22 – 11.47 )		<0.0001	
			Greater BMI	1.07 (1.01 – 1.13 )		0.023	
			History of IBD	1.22 (1.25 – 31.06 )		0.026	
	728 (55)	MODA Definition: ≥ 6 months of disease duration +failed to ≥ 1 cs DMARD (unless contraindicated) and ts/b DMARDs (except for apremilast) with different MOAs + had at least MODA (DAPSA > 14 ) at time of their assessment	Feature of D2T patient	OR (95% CI)		p	- HAQ not captured - Comorbidities documentation based on treatment received - No data for b/ts DMARDs and csDMARD co-administration
			BSA>3%^×^	4.89 (1.89 – 12.64 )		0.001	
			Female Gender	3.03 (1.08 – 8.47 )		0.034	
	393 (49)	MDA Definition: ≥ 6 months of disease duration + failed to ≥ 1 cs DMARD (unless contraindicated) and ≥ 2 ts/b DMARDs (except for apremilast) with different MOAs + were at MDA at time of their assessment	Feature of D2T patient	OR (95% CI)		p	
			BSA>3%^×^	3.28 (1.36 – 7.90 )		0.008	
			History of Axial Disease	2.23 (1.04 – 4.77 )		0.040	

* ≥ 1 of the following: at least moderate disease activity according to validated measures, signs and symptoms of active disease, rapid radiographic progression, or PsA symptoms that lead to reduced QoL.

1 at visit moment

˚ hypertension, dyslipidaemia, metabolic syndrome, DM, CV disease, osteoarthritis, and anxiety/depression being the most common comorbidities ~number of b/tsDMARDs failed

3 at baseline

× at diagnosis

b: biological; BMI: body mass index; BSA: Body Surface Area; CI: Confidence Interval; cs: conventional synthetic; CV: cardiovascular; D2T: difficult to treat; DAPSA: Disease Activity in Psoriatic Arthritis; DM: Diabetes Mellitus; DMARDs: disease modifying anti-rheumatic drugs; FCI: Functional Comorbidity Index; HAQ-DI: Health Assessment Questionnaire Disability Index; HTN: hypertension; IBD: Inflammatory Bowel Disease; MDA: Minimal Disease Activity; MOA: mechanism of action; MODA: Moderate Disease Activity; n: number of patients; nD2T: non difficult to treat; OR: Odds Ratio; p: p-value; PASS: Patient Acceptable Symptom State; PsA: psoriatic arthritis; PsAID: Psoriatic Arthritis Impact of Disease; PtGA: Patient Global Assessment; ts: targeted synthetic; VAS: Visual Analogic Scale.

Afterwards, in 2023, a French study^13^ examining 150 PsA patients, defined D2T PsA as patients who have failed in at least 2 different b/tsDMARDs with different MOAs (Table 1). They showed that D2T PsA patients – compared to not D2T PsA– had more frequent axial involvement and peripheral structural damage at baseline and had more commonly discontinued DMARDs due to poor dermatological control. Philippoteaux et al. also included the “very D2T PsA” category in their study defining it as patients who received at least 2 b/tsDMARDs with different MOA in less than 2 years, being the only study to include a temporal criterion. The three groups, non-D2T, D2T, and very D2T PsA, in a second analysis (Table 2), documented significant differences in PsA disease duration, axial involvement at baseline, peripheral and axial structural damage at baseline, obesity rates and previous csDMARDs and bDMARDs discontinuation frequency. The D2T patients documented longest disease duration, with the not D2T coming second. Compared to the non-D2T, the very D2T group demonstrated higher prevalence of obesity and axial involvement at baseline and the D2T exhibited greater axial and peripheral structural damage at baseline and higher rate of bDMARDs discontinuation due to poor dermatological control.

Thirdly, Vassilakis et al.,^14^ in 2024, retrieved data from the multicentre registry of patients with PsA in Greece and utilised three different D2T PsA definitions (Table 1). According to their main definition, D2T patients had at least 6 months of disease duration, in which they failed at least 1 cs DMARD (unless contraindicated) and at least 2 ts/b DMARDs (except for apremilast) with different MOAs and had either at least MODA (DAPSA >14) and/or were not in MDA at the time of their assessment. Enrolling 467 patients in this analysis, BSA >3%, higher BMI and history of IBD was proven to be associated with D2T. In their second analysis, defining D2T as patients with at least 6 months of disease duration, failure to at least 1 cs DMARD (unless contraindicated) and at least 2 ts/b DMARDs (except for apremilast) with different MOAs and at assessment time with at least MODA (DAPSA >14), they included 728 patients and documented more frequent BSA>3%, and female gender in D2T. Lastly, there was another definition suggested indicating that D2T patients had at least 6 months of disease duration, failed to at least 1 cs DMARD (unless contraindicated) and at least 2 ts/b DMARDs (except for apremilast) with different MOAs and were at Minimal Disease Activity at the time of their assessment. 393 patients were included, and it showed that BSA>3% and history of axial disease were at higher prevalence in D2T than in non-D2T.

**Table 2.** Philippoteaux et al.^13^ findings with the inclusion of “very D2T PsA” category.

**Characteristics**	**Very D2T PsA (n=17)**	**D2T PsA (n=29)**	**nD2T PsA (n=101)**	**Global p-value**	**p-value Very D2T PsA vs nD2T**	**p-value Very D2T PsA vs D2T PsA**	**p-value D2T PsA vs nD2T**
PsA duration (years median)	8	18	11	0.008	0.33	0.006	0.043
Axial involvement^×^	58.8%	34.5%	26%	0.025	0.020	0.32	1.00
Structural damage(peripheral and axial)^×^	68.8%	80%	50%	0.016	0.49	1.00	0.021
Peripheral structural damage	50%	72%	41.8%	0.026	1.00	0.46	0.021
Obesity	64.7%	31%	29.7%	0.018	0.015	0.079	1.00
Previous csDMARDs	70.6%	96.6%	22.2%	0.042	1.00	0.061	0.062
bDMARDS discontinuation due to poor dermatological control	23.5%	53.6%	18.8%	<0.001	1.00	0.17	<0.001

× at baseline.

b: biological; cs: conventional synthetic; DMARDs: Disease Modifying Anti-Rheumatic Drugs; D2T: difficult to treat; nD2T: not difficult to treat; PsA: Psoriatic Arthritis.

## DISCUSSION

Defining D2T PsA has been deemed of high importance. A clear definition will allow for more targeted, relevant and applicable clinical trials. It will ensure the development of effective treatment strategies, tailored guidelines, combination therapies and novel drugs. By providing deeper knowledge of D2T patients’ individual characteristics and predictors, timely identification of potential D2T patients will also be allowed, as well as better monitoring and overall optimal care.

The effort to define D2T PsA has gained momentum following the establishment of criteria for D2T RA. D2T concept was initially coined to describe RA patients who still demonstrated signs of active or progressive disease, despite treatment with csDMARDs and at least 2 different b-ts-DMARDs with different MOA.^11^ After this and stemming from the fact that PsA’s complexity may be even greater than RA’s, given the heterogeneity in clinical expression of the disease, as well as the co-occurrence of extra-musculoskeletal manifestations and various comorbidities, expert panels and task forces have been put forth by the PsA community to establish consensus definitions for D2T PsA. In a recent systematic literature review conducted to feed the GRAPPA project for D2T, it was evident that over the past years, many different definitions have been suggested, varying on the number of failed cs-, b-, and ts-DMARDs.^1^

Thus far, only few studies have been published in the field, examining this issue. From this current narrative review, it seems that certain PsA characteristics have already proven to be linked with D2T status. Greater extent of psoriasis seems to be more frequent in these patients.^12,14^ along with axial involvement.13,14 Moreover, obesity,^12,14^ female gender,^14^ and various comorbidities, such as IBD, depression and fibromyalgia, appear to be poor predisposing factors for disease management and treatment goal achievement.^12,14^

Apparently, characteristics found more commonly in D2T lead to exclusion from use of some therapeutic agents or to indication of specific ones, resulting in diminution of the available arsenal of drugs and of the available escalation strategies, rendering management of disease even more problematic. Characteristically, csDMARDs are not effective in axial involvement and anti-IL-17 or etanercept are contraindicated in active IBD disease.^8,15^ On the other hand, in severe skin involvement IL-12/23, IL-23, or IL-17 inhibitors are preferred and in co-morbid severe uveitis or IBD, monoclonal antibodies against TNF are highly recommended.^16^

Furthermore, females and people with higher BMIs have been proven to demonstrate higher disease activity. In a recent review that included many studies on the effect of gender on PsA, it was shown that women demonstrated greater functional disability and reported worse QoL than men. They also seem to score higher on disease activity scores and patient-reported outcomes (PROs) and they were less likely to achieve a state of MDA.^17^ Similarly, obesity has been documented to have a poor impact on disease activity and treatment outcomes in inflammatory arthritis, such as rheumatoid, psoriatic and axial spondyloarthritis. Obese patients demonstrate low treatment target achieving rate, such as MDA, and are predisposed to poor response to Tumour Necrosis Factor inhibitors.^18^ Depression and fibromyalgia are also factors that appear frequently in conjunction with D2T status. The aetiologic relationship of depression and PsA appears to be bidirectional, figuring from the fact that depressive mood is associated with increased inflammatory markers and inflammation has been associated with depression. Demographics (such as female gender), pain, fatigue, physical disability, social problems, co-morbidities and treatment have all been hypothesised to affect mental health in PsA patients.^3^ Depressive symptomatology influences adherence to treatment, as well as a patient’s perceptive of QoL, and it results in a distorted image in PROs, altogether minimising the chance of MDA achievement and maintenance. In a recent analysis from Gialouri et al.,^3^ co-morbid depression was proven to be associated with a higher number of affected joints, higher inflammatory markers, lower QoL and worse disease activity scores and PROs at assessment time. Commonly co-existent with depression, fibromyalgia (FM), a chronic pain syndrome predominantly characterised by pain, fatigue, sleep disturbance and impaired cognition, has been shown to occur in patients with inflammatory arthritis at a higher rate compared to the general population and specifically in PsA in a 18% (95% CI 13–23%; I^2^=73%).^19^ FM pain mimics enthesitis, making the distinction problematic, whereas all FM symptomatology tends to have an impact on QoL. This co-morbidity has the danger of muddying up disease assessment, much like with depressive symptomatology, resulting in non-reliable disease activity scores and PROs results.^19^

Another field of controversy in PsA are all the indices that can be utilised for measuring disease activity. Not having an appropriate and commonly used measure of remaining disease naturally hinders the process of defining D2T. There are many in use, each with its own strengths and weaknesses in assessing activity, but there is not one single PsA activity index that is ideal for the everyday clinical practice, encompassing all the multiple combinations of disease manifestations, characteristics and co-morbidities, while being as objective as possible.^20^

To add another level of complexity, it has been suggested that two distinct, different terms should be used for PsA patients that do not achieve optimal therapeutic outcomes. On the one hand, the term refractory-to-treatment PsA, refers to patients that exhibit persistent inflammation, due to immunological reasons, despite multiple treatments. On the other hand, the term D2T PsA refers to individuals whose pre-existent clinical characteristics result in difficulty in management and bad treatment outcomes. This distinction seems critical, considering that these two groups of people are resistant to treatment via very different mechanisms and so should be studied, managed and monitored accordingly.^21^

Finally, the potential need for inclusion of temporal criteria in the definition of D2T PsA should be taken into account. Considering that the disease is not stationary, a person with a D2T profile, may just as well get better after a longer period of therapy or with a change in the used medication, especially with the novel drugs that are now available, reverting to a not D2T status, or accordingly, a patient that has been having the desired treatment outcomes could eventually fail to respond to treatment, gaining the D2T status.^14,21^ It should be considered whether someone that was once considered D2T is D2T for life, even after amelioration of disease, or if the characterisation is only for a moment in time. A time period criterion was also discussed in EULAR’s Task Force that enforced the D2T RA, but not all members supported its inclusion.^11^ In a review about D2T RA that provided further consideration upon its definition, the importance of monitoring patients that where at some point considered as D2T but now have effective disease control is considered, stressing that they would be at risk of reversion and highlighting that an approach not concerning only a single point of disease activity could be useful.^22^

In conclusion, it is evident that despite the plethora of therapeutic regimes available for PsA and the efforts for a more tailored approach, personalised management is still inadequate and there is unmet need in the field due to the complexity of the disease.^16^ Through the understanding of the D2T concept, PsA community aims to describe better the patients in need and identify potential treatment gaps facilitating research, that will provide new therapeutic options along with novel strategies.
